# Dignity of a Professional Journal

**Published:** 1892-02

**Authors:** 


### EDITORIAL.

### "DIGNITY OF A PROFESSIONAL JOURNAL."

In the proceedings of the Ohio State Veterinary Medical Association, printed in this number of the JOURNAL, will be found a reference to some of the advertisements contained in the JOURNAL, and a resolution condemning us for advertising certain preparations, specifically those of Spelterine and Sanitas Companies.

THE JOURNAL OF COMPARATIVE MEDICINE never has received the advertisements of any matter, the use of which its Editors do not consider perfectly legitimate, and has always refused to make editorial comment in its reading pages upon any advertisement. In this it stands far above the immense majority of the Veterinary Journals of Europe, and of the Journals of Human Medicine in both America and Europe, which receive advertisements of preparations, the contents of which are absolutely unknown, and which inflict their readers with advertising matter among their scientific articles.

THE JOURNAL OF COMPARATIVE MEDICINE was not founded for the purpose of making money, and one year cost the editors $1,500 out of their own pockets to continue it. It was continued, because the intelligent Veterinarians of the country need a progressive, active Journal, looking to the good of the profession in all parts of the land, and because, if it had changed hands at that time, it would have become a low grade organ. Although the JOURNAL has today a larger circulation, in the United States and Canada, than that of *all* other Veterinary Journals in all languages added together, it does not pay the editors for the time required to edit it.

If the veterinarians of the country wish to aid in keeping up the "dignity of a professional journal" let them observe some of the following rules :

I. Pay for the JOURNAL promptly. (Several hundred subscribers will please remit for one, two or more years arrears.)

II. Notify us of change of address, and do not complain, a year after leaving a place, that the JOURNAL has not been received.

III. Instruct your families that when you die, if they do not want the JOURNAL, that they shall not take it from the post office, but shall notify us at once, and not two years afterwards.

IV. Write your communications as they should appear and do not abbreviate half the words, so that we must practically re-write the whole of them.

V. Send in matter promptly, and not several months after a meeting has taken place, or when what would be news at the time has become old and useless.

